# Interim 2022/23 influenza vaccine effectiveness: six European studies, October 2022 to January 2023

**DOI:** 10.2807/1560-7917.ES.2023.28.21.2300116

**Published:** 2023-05-25

**Authors:** Esther Kissling, Marine Maurel, Hanne-Dorthe Emborg, Heather Whitaker, Jim McMenamin, Jennifer Howard, Ramona Trebbien, Conall Watson, Beth Findlay, Francisco Pozo, Amanda Bolt Botnen, Ciaran Harvey, Angela Rose, European IVE group, Members of the European IVE group, Arne Witdouck, Benedicte Delaere, Benédicte Lissoir, Caroline Wylock, Catherine Sion, Cyril Barbezange, Door Jouck, Els Van Nedervelde, Evelyn Petit, François Dufrasne, Isabelle Thomas, Koen Magerman, Lucie Seyler, Marc Bourgeois, Marc Hainaut, Marieke Bleyen, Marijke Reynders, Melissa Vermeulen, Nathalie Bossuyt, Nicolas Dauby, Sarah Denayer, Sebastien Fierens, Siel Daelemans, Thomas Demuyser, Virgini Van Buggenhout, Xavier Holemans, Vesna Višekruna Vučina, Maja Ilić, Goranka Petrović, Ivan Mlinarić, Sanja Kurečić Filipović, Bernard Kaić, Iva Pem Novosel, Ivana Ferenčak, Irena Tabain, Katica Čusek Adamić, Mirjana Lana Kosanović Ličina, Danijela Lakošeljac, Ivana Mihin Huskić, Diana Nonković, Jens Nielsen, Noémie Sève, Ana-Maria Vilcu, Caroline Guerrisi, Thierry Blanchon, Titouan Launay, Alessandra Falchi, Shirley Masse, Sylvie van der Werf, Vincent Enouf, Bruno Lina, Martine Valette, Anthony Nardone, Ruoran Li, Marlena Kaczmarek, Nathalie Nicolay, Sabrina Bacci, Silke Buda, Luise Goerlitz, Kristin Tolksdorf, Ute Preuss, Ralf Duerrwald, Marianne Wedde, Barbara Biere, Janine Reiche, Gergő Túri, Judit Krisztina Horváth, Beatrix Oroszi, Katalin Kristóf, Lisa Domegan, Joan O’Donnell, Róisín Duffy, Adele McKenna, Charlene Bennett, Jeff Connell, Joanne Moran, Michael Joyce, Ligita Jančorienė, Birutė Zablockienė, Ieva Kubiliūtė, Fausta Majauskaitė, Rolandas Zabockis, Goda Šlekytė, Giedrė Cincilevičiūtė, Auksė Mickienė, Monika Kuliešė, Roberta Vaikutytė, Stephen Abela, Aušra Džiugytė, Maria-Louise Borg, John-Paul Cauchi, Tanya Melillo, Adam Meijer, Marit de Lange, Frederika Dijkstra, Mariëtte Hooiveld, Rianne van Gageldonk, Ana Paula Rodrigues, Ausenda Machado, Irina Kislaya, Verónica Gomez, Aryse Melo, Camila Henriques, Inês Costa, Licínia Gomes, Miguel Lança, Nuno Verdasca, Raquel Guiomar, Mihaela Lazar, Maria Elena Mihai, Alina Ivanciuc, Catalina Pascu, Iulia Bistriceanu, Sorin Dinu, Mihaela Oprea, Odette Popovici, Isabela Loghin, Elena Duca, Mihaela Catalina Luca, Carmen Mihaela Dorobat, Corneliu-Petru Popescu, Gratiela Tardei, Alexandru Marin, Alma-Gabriela Tudor, Simin-Aysel Florescu, Emanoil Ceausu, Rodica Popescu, Olivia Timnea, Adrian Jidovu, Virtudes Gallardo García, Irene Pedrosa Corral, Silvia Martínez, Ana Milagro, Ana Fernández Ibañez, Marta Huerta Huerta, Jordi Reina, Jaume Giménez, Nieves López González-Coviella, Eva Rivas Wagner, Luis Viloria, Tomás Vega Alonso, José Eugenio Lozano Alonso, María Socorro Fernández Arribas, Ana Martínez, Luca Basile, Francesc Botella Quijal, Aurora López Maside, Ana Sofía Lameiras Azevedo, Juan Antonio Linares Dopido, Cecilia Gordillo, Olaia Pérez Martínez, Rosa María García Álvarez, Luis García Comas, Mercedes Rumayor, María Isabel Barranco, Judith Huete Obispo, Ana Isabel Rivas Pérez, Violeta Ramos Marín, Daniel Castrillejo Pérez, Sergio Román Soto, Gloria Pérez-Gimeno, Clara Mazagatos, Amparo Larrauri, Iván Martínez-Baz, Itziar Casado, Jesús Castilla, Aitziber Echeverría, Nerea Egüés, Guillermo Ezpeleta, Ana Navascués, Ana Miqueleiz, Carmen Ezpeleta, Neus Latorre-Margalef, Åsa Wiman, Annasara Carnahan, Katie Hassell, Julia Stowe, Hongxin Zhao, Catherine Quinot, Nick Andrews, Nick Richardson, Katja Hoschler, Angie Lackenby, Catherine Thompson, Maria Zambon, Chris Robertson, Josie Evans, Naoma William, Mark Hamilton

**Affiliations:** 1 Epiconcept, Paris, France; 2 Department of Infectious Disease Epidemiology and Prevention, Statens Serum Institut, Copenhagen, Denmark; 3 UK Health Security Agency, London, United Kingdom; 4 Public Health Scotland, Glasgow, United Kingdom; 5 Department of Virus and Microbiological Special diagnostics, National Influenza Center, Statens Serum Institut, Copenhagen, Denmark; 6 National Centre for Microbiology, National Influenza Reference Laboratory, WHO-National Influenza Centre, Institute of Health Carlos III, Madrid, Spain; 7 CIBER de Epidemiología y Salud Pública (CIBERESP), Institute of Health Carlos III, Madrid, Spain; 8 European Influenza Vaccine Effectiveness (IVE) group members are listed below

**Keywords:** influenza, vaccine effectiveness, multicentre study, test-negative design, Europe

## Abstract

**Background:**

Between October 2022 and January 2023, influenza A(H1N1)pdm09, A(H3N2) and B/Victoria viruses circulated in Europe with different influenza (sub)types dominating in different areas.

**Aim:**

To provide interim 2022/23 influenza vaccine effectiveness (VE) estimates from six European studies, covering 16 countries in primary care, emergency care and hospital inpatient settings.

**Methods:**

All studies used the test-negative design, but with differences in other study characteristics, such as data sources, patient selection, case definitions and included age groups. Overall and influenza (sub)type-specific VE was estimated for each study using logistic regression adjusted for potential confounders.

**Results:**

There were 20,477 influenza cases recruited across the six studies, of which 16,589 (81%) were influenza A. Among all ages and settings, VE against influenza A ranged from 27 to 44%. Against A(H1N1)pdm09 (all ages and settings), VE point estimates ranged from 28% to 46%, higher among children (< 18 years) at 49–77%. Against A(H3N2), overall VE ranged from 2% to 44%, also higher among children (62–70%). Against influenza B/Victoria, overall and age-specific VE were ≥ 50% (87–95% among children < 18 years).

**Conclusions:**

Interim results from six European studies during the 2022/23 influenza season indicate a ≥ 27% and ≥ 50% reduction in disease occurrence among all-age influenza vaccine recipients for influenza A and B, respectively, with higher reductions among children. Genetic virus characterisation results and end-of-season VE estimates will contribute to greater understanding of differences in influenza (sub)type-specific results across studies.

**Key public health message**

**What did you want to address in this study?**

Different types (A or B) or subtypes (e.g. A(H3N2), A(H1N1)pdm09)) of influenza viruses exist. In Europe several virus (sub)types have been co-circulating in the 2022/23 influenza season. We wanted to understand how well the influenza vaccine for this season has protected people so far. Because people’s settings, the virus (sub)types they encounter and their age might all influence vaccine effectiveness, these potential factors were considered.

**What have we learnt from this study?**

In primary care, emergency and hospital settings, interim influenza vaccine effectiveness estimations for 2022/23 indicated some protection by the vaccine. Regardless of setting, all-age vaccine effectiveness against influenza A(H3N2) and A(H1N1)pdm09 virus subtypes ranged from 2 to 46%; this was higher for influenza B (≥ 50%). Vaccine effectiveness point estimates in <18-year-old children were higher (49–95%) than adults across all (sub)types.

**What are the implications of your findings for public health?**

While this report presents interim results, the findings support that influenza vaccination should be continued according to national guidelines as the influenza season unfolds. Further characterisations of circulating influenza viruses and updated vaccine effectiveness estimates at the end of the season will enhance the understanding of the protection conferred by the vaccine in a European context, supporting preparation for future seasons.

## Introduction

In European Union (EU) countries and the United Kingdom (UK), seasonal influenza vaccine is recommended for older adults (mainly considered as those aged ≥ 60 years or ≥ 65 years, depending on the country) and those at increased risk of influenza complications and severe disease (e.g. those with chronic conditions) [1]. Moreover, routine childhood influenza vaccination programmes have been introduced in some World Health Organization (WHO) European Region countries, including in the UK since 2013/14, in Ireland since 2020/21, and in Denmark in 2–6-year-olds only, since 2021/22 [2,3].

The WHO recommended that the 2022/23 northern hemisphere influenza season trivalent influenza vaccine strains to be included in egg-based vaccines should be an A/Victoria/2570/2019 (H1N1)pdm09-like virus, an A/Darwin/9/2021 (H3N2)-like virus and a B/Austria/1359417/2017-like virus (B/Victoria lineage). For non-egg-based vaccines (i.e. cell culture- or recombinant-based vaccines), WHO recommended inclusion of an A/Wisconsin/588/2019 (H1N1)pdm09-like virus, an A/Darwin/6/2021 (H3N2)-like virus and a B/Austria/1359417/2021 (B/Victoria lineage)-like virus. In both egg- and cell-culture-based quadrivalent vaccines, WHO recommended to also include a B/Phuket/3073/2013 virus (B/Yamagata lineage) [4].

The influenza season for 2022/23 started early in most of the 53 WHO European Region countries, with activity crossing the epidemic threshold of 10% sentinel specimen positivity in week 45 2022 and high seasonal influenza virus circulation reported from 29 of the 37 influenza-reporting countries by the first week in January 2023 [5]. In primary care sentinel specimens, during the period covered by this study, which goes up to the end of January (week 4) 2023, influenza A(H3N2) subtypes were initially dominant, with influenza A(H1N1)pdm09 subtypes subsequently dominating from week 2 2023, although there was substantial heterogeneity in influenza A subtype distribution by country [6]. Influenza B virus was also reported. For hospitalised patients, (mostly untyped) influenza A viruses were detected in urgent care wards, while specimens from patients with severe acute respiratory illness (SARI) were predominantly influenza A(H1N1)pdm09 [7]. Other respiratory viruses, particularly severe acute respiratory syndrome coronavirus 2 (SARS-CoV-2) and respiratory syncytial virus (RSV) were also co-circulating during the 2022/23 influenza season, the latter at high levels [7].

The European Centre for Disease Prevention and Control (ECDC)’s Vaccine Effectiveness, Burden and Impact Studies (VEBIS) project began measuring influenza vaccine effectiveness (VE) in the 2022/23 season through multicentre studies in primary care and hospital settings. Previously, many VEBIS study sites were included in the Influenza – Monitoring Vaccine Effectiveness in Europe (I-MOVE) network, which measured influenza VE annually from 2008/09 to 2021/22. The UK and Denmark were I-MOVE partners until 2021/22 and have been estimating influenza VE in single-country studies since 2006 and 2009, respectively.

We report interim influenza VE estimates for the 2022/23 season from six studies (four single- and two multi-country), including out-patient (primary care), in-patient (hospital) and emergency care settings, in order to inform measures of influenza prevention and control for the remaining season.

## Methods

### Study setting

**Figure 1:**
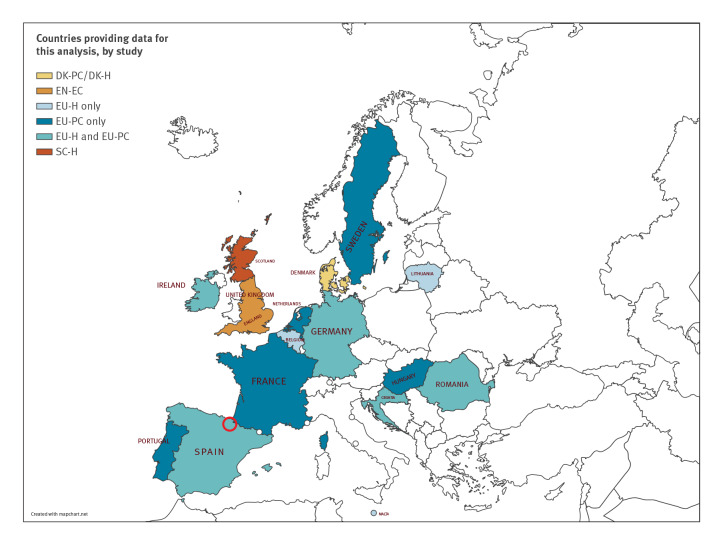
Countries providing interim influenza vaccine effectiveness results, Europe, influenza season 2022/23 (n  = 16)

The two primary care studies were conducted in Denmark (Danish primary care study; DK-PC) and in several EU countries (EU primary care study; EU-PC) through the ECDC VEBIS multi-country network (Table 1). All 10 participating countries in this network had available data for interim analysis; one country, Spain, includes two study sites: Navarra region as one, and 11 other regions combined as the other. The single study at hospital emergency care department level was conducted in England (English emergency care study; EN-EC), with 76% (9,867/13,058) of patients subsequently admitted to hospital. The three studies in the hospital setting were in Denmark (DK-H), Scotland (SC-H) and across several EU countries through the ECDC VEBIS multi-country network (EU-H; Table 1). Seven of 14 participating countries in this network provided data for interim analysis; one country, Spain, has two study sites: one being Navarra and the second comprising 12 other regions combined (Figure 1). A total of 16 European countries (with England and Scotland counted as two countries) contributed data to these interim influenza VE results.

**Table 1:** Summary of methods for the six European interim influenza vaccine effectiveness studies, influenza season 2022/23

Study characteristics	Study					
	DK-PC	EU-PC	EN-EC	DK-H	EU-H	SC-H
Study period	1 Nov 2022 to 29 Jan 2023	3 Oct 2022 to 31 Jan 2023	9 Oct 2022 to 8 Jan 2023	1 Nov 2022 to 29 Jan 2023	10 Oct 2022 to 30 Jan 2023	3 Oct 2022 to 22 Jan 2023
Setting	Non-hospitalised patients^a^	Primary care	Emergency care	Hospital	Hospital	Hospital
Location	DK	HR, FR, DE, HU, IE, NL, PT, RO, ES (Navarra region), ES (11 regions combined, excluding Navarra) and SE	EN	DK	29 hospitals in BE, HR, DE, LT, MT, RO, ES (Navarra region) and ES (12 regions combined, excluding Navarra)	SC
Study design	TND	TND	TND	TND	TND	TND
Data source	Data linkage of Danish Microbiology Database, the Danish Vaccination Register and the Danish National Patient Register	Physicians and laboratory, in some sites data linkage to electronic health records	Data linkage of sentinel laboratory surveillance (Respiratory DataMart), the National Immunisations Management System (NIMS), and the Emergency Care DataSet (ECDS)	Data linkage of Danish Microbiology Database, the Danish Vaccination Register and the Danish National Patient Register	Hospital charts, vaccine registers, interviews with patients, laboratory records	EAVE-II national patient-level dataset, Electronic Communication of Surveillance in SC (ECOSS; all virology testing national database), Rapid Preliminary Inpatient Data (RAPID; Scottish hospital admissions data), National Records of Scotland (NRS; death certification), National Clinical Data Store (NCDS; vaccination events in SC)
Age groups of study population	All ages	All ages^b^	All ages	All ages	All ages^b^	Adults ≥ 18 years old
Case definition for patient recruitment	Sudden onset of symptoms with fever^c^, myalgia and respiratory symptoms^d^	EU ARI^e^ or EU ILI (sudden onset of symptoms AND ≥ 1 of: fever^c^ or feverishness, malaise, headache, myalgia AND ≥ 1 of cough, sore throat, shortness of breath)	Patients with an influenza swab test 14 days before to 7 days after an emergency care visit compatible with influenza infection or its complications	Sudden onset of symptoms with fever^c^, myalgia and respiratory symptoms among patients admitted to hospital for at least 12 hours	EU SARI (hospitalised person with ≥ 1 of fever^c^/feverishness, malaise, headache, myalgia, deterioration of general condition (asthenia, weight loss, anorexia, confusion/dizziness) AND ≥ 1 respiratory symptom (cough, sore throat or shortness of breath) at admission or within 48 hours after admission)	Patients with EU ARI^e^ symptoms and clinician’s judgement that there is an infection^f^ and limited to emergency care where the influenza test occurs 14 days before admission or within 48 hours after admission
Selection of patients	At practitioner's/ clinician's judgement	Systematic	At practitioner's/ clinician's judgement	At practitioner's/ clinician's judgement	Exhaustive (DE, HR, LT, MT, NA, RO) Systematic (BE, ES; some hospitals in BE: exhaustive on either 1 or 2 days per week, depending on workload)	At practitioner's/ clinician's judgement
Vaccine types used nationally or in the study^g,h^	100% QIV (children 2–6 years of age are offered a LAIV as nasal spray)	In the study among controls: among the seven study sites providing information on influenza vaccine brand, the brand was unknown in 19% of vaccinated controls; among the rest, 8% used a QIVc, 2% a LAIV, 12% an egg-propagated trivalent and 78% a QIVe	In the study among controls: ages 2–17 years (90% LAIV, 7% QIVc, 1% QIVe, 2% unknown); ages 18–64 years (74% QIVc, 14% QIVe, 2% QIVr, 5% aQIV, 5% unknown); ages ≥ 65 years (4% QIVc, 1% QIVr, 91% aQIV, 5% unknown)	100% QIV (children 2–6 years of age are offered a LAIV as nasal spray)	In the study among controls: in the only site providing influenza vaccine brand information, 77% were QIV; the remaining 23% unknown (all countries participating use QIV nationally)	22% QIVc; 78% aQIV
Variables of adjustment	Age group, sex^i^, presence of chronic conditions, calendar time as month (Nov–Jan) (if possible, week)	Age (modelled as RCS, age group or linear term depending on analysis), sex^i^, presence of chronic conditions, onset date (RCS) and study site	Age group, region, clinically extremely vulnerable, COVID-19 vaccination status, calendar time as week (spline)	Age group, sex^i^, presence of chronic conditions, calendar time as month (Nov–Jan) (if possible, week)	Age (modelled as RCS, age group or linear term depending on analysis), sex^i^, presence of chronic conditions, time (onset date as RCS or month of swab as categorical term) and study site	Age (spline), sex^i^, number of QCOVID^j^ clinical risk groups (0,1,2,3,4, ≥ 5)^j^, time (days, spline), setting (community or hospital) and deprivation quintile (SIMD)

ARI: acute respiratory infection; aQIV: adjuvanted QIV; BE: Belgium; DE: Germany; DK: Denmark; DK-H: DK hospital study; DK-PC: DK primary care study; EN: England; EN-EC: EN emergency care study; ES: Spain; EU: European Union; EU-H: EU hospital multicentre VEBIS study; EU-PC: EU primary care multicentre VEBIS study; FR: France; GP: general practitioner; HR: Croatia; HU: Hungary; IE: Ireland; ILI: influenza-like illness; LAIV4: quadrivalent live attenuated influenza vaccine; LT: Lithuania; LRI: lower respiratory infection; MT: Malta; NL: Netherlands; PT: Portugal; QIV: quadrivalent inactivated influenza vaccine; QIVc: cell-grown QIV; QIVe: egg-grown QIV; QIVr: recombinant QIV; RCS: restricted cubic spline; RO: Romania; SARI: severe acute respiratory infection; SARS-CoV-2: severe acute respiratory syndrome coronavirus 2; SC: Scotland; SC-H: SC hospital study; SIMD: Scottish Index of Multiple Deprivation; SE: Sweden; TIV: trivalent inactivated influenza vaccine; TND: test-negative design; VE: vaccine effectiveness; VEBIS: VE, Burden and Impact Studies.

^a^ Patients are seen by the GP, but also in emergency care.

^b^ Patients < 6 months of age should have been excluded from the study, however the age group is specified as ‘all ages’ as age in months could not be verified from the data.

^c^ For EU-PC and EU-H, there is no temperature threshold reported for fever, which can be measured or self-reported. For DK-PC and DK-H there is no temperature threshold for fever given in the guidance to practitioners.

^d^ This is the case definition for patient recruitment for all GPs within DK-PC. Sentinel GPs within DK-PC follow the EU-ILI case definition (sudden onset of symptoms, AND ≥1 of: fever >38°C, feverishness, malaise, headache, myalgia AND ≥1 of: cough, sore throat, shortness of breath).

^e^ The EU-ARI definition is sudden onset of symptoms AND ≥ 1 of cough, sore throat, shortness of breath or coryza AND a clinician’s judgement that the illness is due to an infection.

^f^ Varies according to SARS-CoV-2/influenza testing practices by Health Board.

^g^ Vaccines were prepared from egg-propagated vaccine viruses, non-adjuvanted and administered intramuscularly unless otherwise specified.

^h^ Where indicated, vaccine coverage among controls were used as representative of the source population from which the cases arose.

^i^ For all studies ‘sex’ is used in the statistical model for estimating VE as a binary variable: male/female.

^j^ The QCOVID risk groups are defined as the number of generic comorbidity conditions of a patient, and are used as a measure of comorbidity. The list of conditions is found in the study of Clift et al [26].

DK-PC/DK-H: Denmark primary care and hospital studies; EN-EC: England emergency care study; EU-PC/EU-H: European Union primary care-based and hospital-based multi-country VEBIS studies; SC-H: Scotland hospital study; VEBIS: Vaccine Effectiveness, Burden and Impact Studies.

Red circle: Navarra site (EU-PC and EU-H).

### Study design

The methods for all six studies have been described elsewhere [8-12]. All studies used the test-negative design [13], although some differed in how they recruited patients and/or in their data collection (Table 1). Briefly, four studies obtained data through electronic database linkage (DK-H, DK-PC, EN-EC, SC-H) and two predominantly used prospective patient recruitment (EU-PC and EU-H). For primary care studies, nasopharyngeal (or saliva specimens, in France) were collected from patients with influenza-like illness (ILI) or acute respiratory infection (ARI) symptoms. For the emergency care setting, patients were recruited if they had an influenza swab test from 14 days before to 7 days after an emergency care visit compatible with influenza infection or its complications. In the hospital setting, patients admitted with severe ARI (SARI) symptoms were swabbed. In SC-H, all patients entering hospital as an emergency admission and tested for influenza were assumed to have an ARI symptom.

Two studies used an exhaustive or systematic selection of patients to swab/include (EU-PC and EU-H), while physicians' discretion was used to select patients for swabbing in the other four (DK-H, DK-PC, EN-EC and SC-H). Samples were tested by reverse transcription (RT)-PCR for influenza virus detection, type A subtyping and type B lineage determination. We defined cases as patients with positive results by influenza virus (sub)type. We defined controls as those testing RT-PCR negative for influenza virus. Most studies recruited patients among all ages. In SC-H, analyses are restricted to patients aged ≥ 18 years, as not all National Health Service Health Boards in Scotland submit patient level data for vaccination events in children. In EU-H, a few hospitals in some study sites only recruit patients aged ≥ 18 years.

Vaccinated patients were defined as those having had the 2022/23 influenza vaccine at least 14 days before onset of symptoms. Those vaccinated less than 14 days before symptom onset, or with unknown date of vaccination, were excluded. In EN-EC children vaccinated within 20 days were excluded from the analysis to avoid live attenuated influenza vaccine (LAIV)-related infections.

Many study countries (eight from EU-PC; three from EU-H; and Denmark) selected all or a random sample of influenza virus-positive specimens for haemagglutinin genome segment and/or whole genome sequencing, where technically feasible. In SC-H, the selection was based on other criteria (including vaccination status, antiviral use and being from an area with other cases) and cannot be considered a random sample. Sequencing was followed by phylogenetic analysis to determine clade distribution for potential impact on VE. Sequencing results were provided for both studies in Denmark together (DK-PC and DK-H).

### Statistical analysis

We computed VE in each study as one minus the adjusted ratio of the odds of vaccination in cases and controls, as a percentage: VE = (1 − OR_a_) × 100. We applied logistic regression to adjust for measured potential confounding variables (Table 1). We estimated study-specific VE overall and, where possible, by age group and target population (as defined locally in the various studies and study sites) against influenza A and B combined (not in DK-PC or DK-H, as this country decided to only present A and B types separately due to the heterogeneity across influenza type-specific estimates), influenza A overall, A(H1N1)pdm09, A(H3N2), and influenza B. We defined small sample size analyses as those having fewer than 10 cases or controls per parameter. For these, a sensitivity analysis was performed using Firth’s method of penalised logistic regression (PLR) to assess small sample bias [14,15]. We considered a difference of > 10% between the original estimate and that obtained using PLR to be an indication of small sample bias; none of these estimates are shown.

## Results

From 3 October 2022 to 31 January 2023, for the primary care setting we included 6,097 cases and 30,957 controls in the DK-PC study; 3,977 and 10,184 in EU-PC. For the emergency care setting there were 3,760 cases and 9,298 controls. In the hospital setting, there were 1,520 cases and 32,581 controls in DK-H; 488 and 2,620 in EU-H; 4,635 and 29,442 in SC-H.

**Figure 2:**
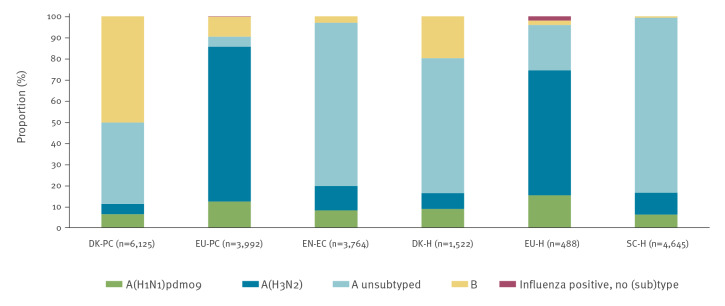
Proportion of influenza virus (sub)type infections, six European studies, interim influenza season 2022/23 (n = 20,536)^a^

DK-H: Denmark hospital study; DK-PC: Denmark primary care study; EC: Emergency care; EN-EC: England emergency care study; EU: European Union; EU-H: EU hospital multicentre VEBIS study; EU-PC: EU primary care multicentre VEBIS study; PC: primary care; SC-H: Scottish hospital study; VEBIS: Vaccine Effectiveness, Burden and Impact Studies.

^a^ Includes 28 A and B co-infections in DK-PC, two in DK-H, four in EN-EC, 10 in EU-PC; includes five A(H1N1)pdm09 and A(H3N2) co-infections in EU-PC and 10 in EU-H.

Overall, 81% (16,604/20,536) of confirmed infections were influenza A virus-positive and 19% (3,913/20,536) were influenza B virus-positive (the remaining 19 (< 1%) being untyped), noting that there were 20,536 infections among 20,477 cases. There were 44 influenza A and B co-infections and 15 influenza A(H1N1)pdm09 and A(H3N2) co-infections. The proportion of subtyped influenza A viruses was 95% in EU-PC, 78% in EU-H, and 17–23% in DK-PC, DK-H, EN-EC and SC-H. Most subtyped influenza A viruses were influenza A(H3N2) (58–85%) in EN-EC, EU-PC, EU-H and SC-H; this subtype comprised 43–46% in DK-PC and DK-H (Figure 2). The proportion of influenza B among all influenza viruses ranged from 1 to 9% in EN-EC, EU-PC, EU-H and SC-H to 20–50% in DK-H and DK-PC (Figure 2).

### All influenza (A and B)

The overall VE estimates for influenza types A and B together were not presented for DK-PC and DK-H due to difference in VE across influenza types.

#### Primary care and emergency care settings

The VE against all influenza among children aged 0–17 years was 55% (95% confidence interval (CI): 31 to 71) in EU-PC; 61% (95% CI: 50 to 70) in EN-EC for those aged 2–17 years. For adults, VE varied between 28% (95% CI: 16 to 38) among those aged ≥ 65 years in EN-EC and 43% (95% CI: 29 to 54) among those aged 18–64 years in EU-PC (Table 2).

**Table 2:** Interim adjusted vaccine effectiveness (VE) against all laboratory-confirmed influenza, influenza A, A(H1N1)pdm09, A(H3N2) and B, by age group, target group for vaccination and by study, six European studies, influenza season 2022/23

Influenza (sub)type and study	Setting	Study population^a^	Cases			Controls			VE^b^	95% CI
			All	Vaccinated	%	All	Vaccinated	%		
**All influenza (A and B)^c^**											
EU-PC	PC	All ages	3,977	275	7	10,184	1,582	16	**44**	34 to 52
		0–17 years	1,679	36	2	2,892	96	3	**55**	31 to 71
		18–64 years	2,067	123	6	5,598	524	9	**43**	29 to 54
		≥ 65 years	231	116	50	1,694	962	57	**33**	8 to 51
		Target group^d^	1,005	220	22	4,112	1,367	33	**38**	24 to 49
EN-EC	EC	All ≥ 18 years	3,046	1,158	38	7,797	3,722	48	**30**	21 to 38
		2–17 years	714	116	16	1,501	389	26	**61**	50 to 70
		18–64 years	1,667	315	19	2,954	798	27	**36**	22 to 48
		≥ 65 years	1,379	843	61	4,843	2,924	60	**28**	16 to 38
EU-H	H	All ages	488	170	35	2,620	1,249	48	**27**	6 to 44
		18–64 years	157	25	16	500	135	27	**17**	−44 to 52
		≥ 65 years	279	143	51	1,597	1,086	68	**29**	4 to 47
		Target group^d^	378	161	43	1,959	1,212	62	**31**	9 to 48
SC-H	H	All ≥ 18 years	4,635	2,317	50	29,442	14,709	50	**29**	24 to 35
		18–64 years	1,987	492	25	10,166	2,561	25	**34**	26 to 42
		≥ 65 years	2,648	1,825	69	19,276	12,148	63	**27**	19 to 34
**Influenza A (all)**											
DK-PC	PC	All ages	3,048	612	20	30,957	9,996	32	**44**	37 to 50
		2–6 years	339	18	5	2,368	399	17	**77**	62 to 86
		0–17 years	898	25	3	8,804	474	5	**61**	40 to 74
		18–64 years	1,702	226	13	14,189	3,065	22	**49**	41 to 56
		≥ 65 years	448	361	81	7,964	6,457	81	**19**	−4 to 37
EU-PC	PC	All ages	3,602	265	7	10,174	1,578	16	**40**	30 to 49
		0–17 years	1,492	35	2	2,890	96	3	**50**	22 to 68
		18–64 years	1,883	116	6	5,592	522	9	**40**	25 to 52
		≥ 65 years	227	114	50	1,692	960	57	**32**	7 to 51
		Target group^d^	943	214	23	4,107	1,363	33	**35**	21 to 47
EN-EC	EC	All ≥ 18 years	2,969	1,152	39	7,797	3,722	48	**29**	20 to 37
		2–17 years	680	114	17	1,501	389	26	**60**	48 to 69
		18–64 years	1,596	312	20	2,954	798	27	**35**	20 to 46
		≥ 65 years	1,373	840	61	4,843	2,924	60	**27**	16 to 38
DK-H	H	All ages	1,221	620	51	32,581	18,513	57	**33**	23 to 42
		2–6 years	49	1	2	708	115	16	**90**	23 to 99
		0–17 years	142	2	1	3,116	145	5	**80**	16 to 95
		18–64 years	356	87	24	8,390	2,316	28	**36**	17 to 50
		≥ 65 years	723	531	73	21,075	16,052	76	**29**	16 to 40
EU-H	H	All ages	468	166	35	2,611	1,245	48	**27**	6 to 44
		18–64 years	146	24	16	498	134	27	**12**	−53 to 50
		≥ 65 years	272	140	51	1,590	1,083	68	**28**	2 to 47
		Target group^d^	363	157	43	1,951	1,208	62	**30**	7 to 47
SC-H	H	All ≥ 18 years	4,601	2,310	50	29,381	14,671	50	**29**	23 to 34
		18–64 years	1,961	489	25	10,141	2,552	25	**34**	25 to 42
		≥ 65 years	2,640	1,821	69	19,240	12,119	63	**26**	18 to 33
**Influenza A(H1N1)pdm09**											
DK-PC	PC	All ages	394	74	19	30,957	9,996	32	**46**	26 to 60
		2–6 years	42	2	5	2,368	399	17	**79**	14 to 95
		0–17 years	122	2	2	8,804	474	5	**77**	6 to 94
		18–64 years	224	36	16	14,189	3,065	22	**35**	6 to 55
		≥ 65 years	48	36	75	7,964	6,457	81	**37**	−22 to 67
EU-PC	PC	All ages^e^	485	55	11	9,569	1,488	16	**28**	0 to 50
		18–64 years	328	26	8	5,261	494	9	**42**	9 to 64
		Target group^d^	168	50	30	3,896	1,285	33	**8**	−39 to 39
EN-EC	EN	All ≥ 18 years	244	94	39	2,357	1,116	47	**26**	−9 to 50
		2–17 years	65	9	14	660	134	20	**49**	−17 to 77
		18–64 years	133	30	23	995	271	27	**21**	−44 to 57
		≥ 65 years	111	64	58	1,362	845	62	**28**	−21 to 57
DK-H	H	All ages	135	64	47	32,581	18,513	57	**34**	1 to 56
		18–64 years	45	13	29	8,390	2,316	28	**22**	−51 to 60
		≥ 65 years	73	50	68	21,075	16,052	76	**42**	5 to 65
SC-H	H	All ≥ 18 years	290	116	40	29,435	14,707	50	**42**	24 to 56
		18–64 years	142	23	16	10,161	2,561	25	**56**	19 to 73
		≥ 65 years	148	93	63	19,274	12,146	63	**31**	−1 to 52
**Influenza A(H3N2)**											
DK-PC^c^	PC	All ages	297	80	27	30,957	9,996	32	**23**	−7 to 45
		18–64 years	163	24	15	14,189	3,065	22	**36**	1 to 59
EU-PC	PC	All ages^e^	2,913	193	7	9,879	1,544	16	**44**	32 to 54
		0–17 years	1,291	25	2	2,874	95	3	**62**	37 to 78
		18–64 years	1,455	86	6	5,379	512	10	**39**	22 to 53
		≥ 65 years	167	82	49	1,626	937	58	**39**	11 to 57
		Target group^d^	719	150	21	3,961	1,333	34	**41**	26 to 54
EN-EC	EC	All ≥ 18 years	288	104	36	2,357	1,116	47	**37**	12 to 55
		2–17 years	144	17	12	660	134	20	**70**	46 to 84
		18–64 years	145	28	19	995	271	27	**42**	−5 to 68
		≥ 65 years	111	76	68	1,362	845	62	**42**	10 to 62
DK-H	H	All ages	115	65	57	32,581	18,513	57	**2**	−53 to 37
EU-H	H	All ages^f^	288	120	42	2,575	1,242	48	**27**	1 to 46
		≥ 65 years	191	103	54	1,574	1,080	69	**28**	−3 to 49
		Target group^d^	238	114	48	1,929	1,205	62	**26**	−3 to 47
SC-H	H	All ≥ 18 years	479	152	32	29,381	14,703	50	**32**	16 to 45
		18–64 years	188	42	22	10,164	2,559	25	**36**	7 to 56
		≥ 65 years	291	203	70	19,272	12,144	63	**33**	12 to 49
**Influenza B**											
DK-PC	PC	All ages	3,077	94	3	30,957	9,996	32	**85**	82 to 88
		2–6 years	270	3	1	2,368	399	17	**95**	85 to 99
		0–17 years	1,511	11	1	8,804	474	5	**90**	82 to 95
		18–64 years	1,532	62	4	14,189	3,065	22	**86**	82 to 89
		≥ 65 years	34	21	62	7,964	6,457	81	**66**	33 to 83
EU-PC	PC	All ages^e^	368	10	3	8,601	1,388	16	**64**	32 to 83
		0–17 years	190	1	1	2,638	93	4	**90**	52 to 100
		18–64 years	177	8	5	4,553	448	10	**51**	1 to 79
EN-EC	EC	All ≥ 18 years	80	6	8	7,797	3,722	48	**78**	44 to 92
		2–17 years	35	2	6	1,501	389	26	**88**	47 to 97
		18–64 years	73	3	4	2,954	798	27	**84**	43 to 95
DK-H	H	All ages	301	39	13	32,581	18,513	57	**73**	61 to 82
		2–6 years	35	1	3	708	115	16	**87**	6 to 98
		0–17 years	132	1	1	3,116	145	5	**89**	19 to 98
		18–64 years	125	11	9	8,390	2,316	28	**78**	59 to 88
		≥ 65 years	44	27	61	21,075	16,052	76	**58**	22 to 77
SC-H	PC	All ≥ 18 years	34	7	21	29,410	14,702	50	**50**	−36 to 82

CI: confidence interval; DK-H: Denmark hospital study; DK-PC: Denmark primary care study; EC: Emergency care; EN-EC: England emergency care study; EU: European Union; EU-H: EU hospital multicentre VEBIS study; EU-PC: EU primary care multicentre VEBIS study; PC: primary care; SC-H: Scottish hospital study; VE: vaccine effectiveness; VEBIS: VE, Burden and Impact Studies.

^a^ Age-specific or target group-specific VE was not included for overall or (sub)type-specific VE in some study sites, where sample size did not allow estimation of VE. Additionally for EU-PC: three study sites with < 10 A(H1N1)pdm09 cases were excluded from A(H1N1)pdm09 VE analysis (11 cases); two study sites with < 10 A(H3N2) cases were dropped from A(H3N2) VE analysis (8 cases); five study sites with < 10 B cases were not included in B VE analysis (8 cases).

^b^ For details of adjustment variables, see Table 1.

^c^ All influenza (A and B) estimates were not presented for DK-PC and DK-H due to difference in VE across influenza types.

^d^ Groups targeted by seasonal influenza vaccination as defined locally in the studies and study sites.

^e^ Three study sites with < 10 A(H1N1)pdm09 cases were excluded from A(H1N1)pdm09 VE analysis (11 cases); two study sites with < 10 A(H3N2) cases were dropped from A(H3N2) VE analysis (8 cases); five study sites with < 10 B cases were not included in B VE analysis (8 cases).

^f^ Two study sites with < 10 influenza A(H3N2) cases were dropped from A(H3N2) VE analysis (10 cases).

#### Hospital inpatient settings

For older adults (aged ≥ 65 years), VE against all laboratory-confirmed hospitalised influenza was 29% (95% CI: 4 to 47) in EU-H and 27% (95% CI: 19 to 34) in SC-H. In the EU-H target group for influenza vaccination, VE was 31% (95% CI: 9 to 48).

### Influenza A overall

For all ages and regardless of setting, VE against influenza A ranged from 27% (95% CI: 6 to 44) in EU-H to 44% (95% CI: 37 to 50) in DK-PC.

#### Primary care and emergency care settings

Among 18–64-year-olds, VE against laboratory-confirmed influenza A ranged from 35% (20 to 46) in EN-EC to 49% (41 to 56) in DK-PC. The VE against influenza A among people ≥ 65 years old ranged from 19% (95% CI: −4 to 37) in DK-PC to 32% (95% CI: 7 to 51) in EU-PC. In children < 18 years old, VE ranged from 50% (95% CI: 22 to 68) in EU-PC (0–17-year-olds), to 77% (95% CI: 62 to 86) in DK-PC (2–6-year-olds) (Table 2).

#### Hospital inpatient settings

For all ages, VE against laboratory-confirmed hospitalised influenza A ranged from 27% (95% CI: 6 to 44) in EU-H to 33% (95% CI: 23 to 42) in DK-H. For children, VE was between 80% (95% CI: 16 to 95) in those aged 0–17 years in DK-H and 90% (95% CI: 23 to 99) among those aged 2–6 years in DK-H. For adults aged 18–64 years, VE ranged from 12% in EU-H (95%CI: −53 to 50) to 36% in DK-H (95%CI: 17 to 50). For adults ≥ 65 years of age, VE ranged between 26% (95% CI: 18 to 33) in SC-H and 29% (95% CI: 16 to 40) in DK-H.

### Influenza A(H1N1)pdm09

For all ages and regardless of setting, VE against A(H1N1)pdm09 ranged from 28% (95% CI: 0 to 50) in EU-PC to 46% (95% CI: 26 to 60) in DK-PC.

#### Primary care and emergency care settings

The VE against laboratory-confirmed influenza A(H1N1)pdm09 among children < 18 years of age was 49% (95% CI: −17 to 77) in EN-EC (2–17 years) and 77% (95% CI 6 to 94) in DK-PC (0–17 years). Among adults < 65 years old, VE ranged between 21% (95% CI: −44 to 57) in EN-EC and 42% (95% CI: 9to 64) in EU-PC. VE for people ≥ 65 years old was 28% (95% CI: −21 to 57) in the EN-EC study and 37% (95% CI: −22 to 67) in the DK-PC study. Target groups in the EU-PC had VE of 8% (95% CI: −39 to 39) against influenza A(H1N1)pdm09.

#### Hospital inpatient settings

For hospitalised patients aged 18–64 years, VE against A(H1N1)pdm09 was 22% (95% CI: −51 to 60) in DK-H and 56% (95% CI: 19 to 73) in SC-H. Among adults aged ≥ 65 years old, VE was 31% (95% CI: −1 to 52) in SC-H and 42% (95% CI: 5 to 65) in the DK-H study (Table 2). Sample size was too small to calculate VE estimates against influenza A(H1N1)pdm09 for the EU-H study.

#### Virological results

Among the 175 A(H1N1)pdm09 viruses sequenced, all but one 99% (n = 174) belonged to genetic clade 6B.1A.5a.2 (Table 3), the same as the vaccine virus. Among these 6B.1A.5a.2 viruses, 59/80 (74%) and 57/74 (77%) were A/Sydney/5/2021 A(H1N1)pdm09-like viruses in EU-PC and DK-H/DK-PC, respectively. These viruses had undergone the K54Q, A186T, Q189E, E224A, R259K and K308R amino acid changes compared with the vaccine virus A/Victoria/2570/2019. All 20 SC-H, 23% (n = 17) of the 74 DK-H/DK-H and 26% (n = 21) of the 81 EU-PC viruses were A/Norway/25089/2022 A(H1N1)pdm09-like viruses, characterised by the amino acid mutations P137S, K142R, D260E and T277A, compared to the vaccine strain. Study-specific virological data were not available in EN-EC, but from English national virological surveillance data, ca 80% of influenza A(H1N1)pdm09 viruses were A/Norway/25089/2022-like and ca 20% of viruses are A/Sydney/5/2021-like (Maria Zambon, personal communication, March 2023).

**Table 3:** Influenza viruses characterised by clade, amino acid substitutions and study site, six European studies, interim influenza season 2022/23 (n = 806)

Characterised viruses	Clade	DK-H/DK-PC^a^		EU-PC^b^		EU-H		SC-H	
		n	%	n	%	n	%	N	%
Influenza A(H1N1)pdm09 (n = 175)		n = 74		n = 81		n = 0		n = 20	
A/Guangdong Maonan/SWL1536/2019	6B.1A.5a.1	0	NC	1	1	0	NC	0	NC
A/Victoria/2570/2019	6B.1A.5a.2	0	NC	0	NC	0	NC	0	NC
A/Sydney/5/2021^c^	6B.1A.5a.2	57	77	59	73	0	NC	0	NC
A/Norway/25089/2022^d^	6B.1A.5a.2	17	23	21	26	0	0	20	NC
Influenza A(H3N2) (n = 570)		n = 93		n = 444		n = 18		n = 15	
A/Denmark/3264/2019	3C.2a1b.1a	0	NC	0	NC	0	NC	0	NC
A/Cambodia/e0826360/2020	3C.2a1b.2a.1	0	NC	0	NC	0	NC	0	NC
A/Darwin/9/2021	3C.2a1b.2a.2	8	9	0	NC	0	NC	0	NC
A/Slovenia/8720/2022^e^	3C.2a1b.2a.2	40	43	113	25	4	NC	0	NC
A/Bangladesh/4005/2020^f^	3C.2a1b.2a.2	45	48	331	75	14	NC	15	NC
Group (i) S156H + others	NA	45	NC	296	89	13	NC	0	NC
Group (ii) D53N + others	NA	0	NC	35	11	1	NC	0	NC
Influenza B/Victoria (n = 82)		n = 22		n = 60		n = 0		n = 0	
B/Washington/02/2019	V1A.3	0	NC	0	NC	0	NC	0	NC
B/Netherlands/11267/2022	V1A.3	0	NC	0	NC	0	NC	0	NC
B/Cote d'Ivoire/948/2020	V1A.3a.1	0	NC	0	NC	0	NC	0	NC
B/Austria/1359417/2021	V1A.3a.2	22	NC	60	100	0	NC	0	NC

DK-H: Denmark hospital study; DK-PC: Denmark primary care study; EU-H: European Union hospital multicentre VEBIS study; EU-PC: European Union primary care multicentre VEBIS study; NA: not applicable; NC: not calculated (percentages not shown where denominators < 60); SC-H: Scotland hospital study; VEBIS: Vaccine Effectiveness, Burden and Impact Studies.

^a^ DK-H and DK-PC samples are combined.

^b^ At time of writing, issues between linkage of epidemiological record identification (ID) numbers and sequencing ID numbers resulted in a low proportion of viruses included from two study sites within EU-PC.

^c^ Harbouring the K54Q, A186T, Q189E, E224A, R259K and K308R amino acid mutations compared with the vaccine virus A/Victoria/2570/2019.

^d^ Harbouring the A/Sydney/5/2021-like amino acid changes, and additionally P137S, K142R, D260E and T277A amino acid mutations compared with the vaccine virus A/Victoria/2570/2019.

^e^ Harbouring the D53G, D104G and K276R amino acid mutations compared with the vaccine virus A/Darwin/9/2021.

^f^ Harbouring the S156H amino acid mutation among others or the D53N amino acid mutation among others compared with the vaccine virus A/Darwin/9/2021.

### Influenza A(H3N2)

For all ages and regardless of setting, VE against A(H3N2) ranged from 2% (95% CI: −53 to 37) in DK-H to 44% (95% CI: 32 to 54) in EU-PC.

#### Primary care and emergency care settings

For children aged 0–17 years in EU-PC, VE against influenza A(H3N2) was 62% (95% CI: 37 to 78). For children aged 2–17 years in the EN-EC, VE was 70% (95% CI: 46 to 84). For those aged 18–64 years, VE was between 36% (95% CI: 1 to 59) in DK-PC and 42% (95% CI: −5 to 68) in EN-EC. VE was 39% (95% CI: 11 to 57) in EU-PC and 42% (95% CI: 10 to 62) in EN-EC in those aged ≥ 65 years (Table 2).

#### Hospital inpatient settings

VE among hospitalised patients of all ages was 2% (95% CI: −53 to 37) in DK-H, 27% (95% CI: 1 to 46) in EU-H and 32% (95% CI: 16 to 45) in SC-H, the latter among those aged ≥ 18 years. Among adults aged ≥ 65 years, VE against influenza A(H3N2) was 28% (95% CI: −3 to 49) in EU-H and 33% (95% CI: 12 to 49) in SC-H (Table 2).

#### Virological results

Of the 570 influenza A(H3N2) viruses sequenced, all belonged to the same clade as the vaccine strain (3C.2a1b.2a.2). In DK-PC/DK-H, 43% (40/93), in EU-PC 25% (113/444), and in EU-H 22% (4/18) of the viruses belonged to A/Slovenia/8720/2022-like viruses, harbouring the specific amino acid mutations D53G, D104G and K276R, while no such viruses were sequenced in SC-H. In DK-PC/DK-H 48% (45/93), in EU-PC 75% (331/444), in EU-H 78% (14/18) and in SC-H 100% (15/15) belonged to A/Bangladesh/4005/2020-like viruses. Among these, where known, 100% (n = 45) in DK-PC/DK-H, 89% (296/331) in EU-PC and 93% (13/14) in EU-H belonged to group (i), harbouring the S156H amino acid mutation among others. In EU-PC 11% (35/331) and in EU-H 7% (1/14) belonged to group (ii), harbouring the amino acid mutation D53N among others. In DK-PC/DK-H 9% (8/93) belonged to the northern hemisphere vaccine strain A/Darwin/9/2021-like virus (Table 3).

### Influenza B

#### Primary care and emergency care settings

Among children, VE against laboratory-confirmed influenza B ranged from 88% (95% CI: 47 to 97) in EN-EC among those aged 2–17 years to 95% (95% CI: 85 to 99) in DK-PC for those aged 2–6 years. For those aged 18–64 years, VE was 51% in EU-PC (95% CI: 1 to 79) and 86% (95% CI: 82 to 89) in DK-PC. The VE among those aged ≥ 65 years was 66% (95% CI: 33 to 83%) in DK-PC (Table 2).

#### Hospital settings

The VE against influenza B was 50% (95% CI: −1 to 52) in SC-H among those aged ≥ 18 years. In DK-H, the VE was 78% (95% CI: 59 to 88) among those aged 18–64 years and 58% (95% CI: 22 to 77) among those aged ≥ 65 years. Among children aged 0–17 years, the VE in DK-H was 89% (95% CI: 19 to 98) (Table 2). Sample size was too small to calculate VE estimates against influenza B for the EU-H study.

#### Virological results

Of the 82 influenza B/Victoria viruses sequenced, all belonged to the V1A.3a.2 clade, represented by B/Austria/1359417/2021, which is also the vaccine virus (Table 3).

### Sensitivity analyses

Results with small sample sizes were subject to sensitivity analyses, most of which gave similar results (absolute difference < 10%). Results from the three estimates with absolute difference ≥ 10% (evidence of small sample bias) were not presented.

## Discussion

In six well-established influenza studies across Europe during the 2022/23 influenza season, interim VE against influenza A (all subtypes) (all ages; primary care, emergency care and hospital settings) ranged from 27% to 44%. All interim VE against influenza B was ≥ 50%, among overall and age-stratified estimates. The proportions of influenza A and B and influenza A subtypes circulating differed by country and setting.

Influenza A (all subtypes) point estimates for VE were higher among children (50–90%), compared with adults (12–49%). Against influenza A(H1N1)pdm09, VE point estimates among all ages ranged from 28% to 46%. The VE point estimates were higher among children at 49% and 77% in EN-EC and DK-PC, respectively (21–56% among those aged 18–64 years). VE against influenza A(H3N2) ranged from 2 to 44% among all ages (36–42% among 18–64-year-olds). Children had higher VE point estimates at 62–70%. Against laboratory-confirmed influenza B, VE in children < 18 years old was between 88 and 90% (87–95% in those aged 2–6 years old).

The proportion of subtyped influenza A viruses varied by study site (between 17% and 95%). While the lack of subtyping may have affected the precision of subtype-specific estimates, descriptive analyses at study site level indicated that those subtyped are likely to belong to a representative sample of all viruses.

In the EN-EC and EU-PC studies, for which the end-of-season 2021/22 influenza A(H1N1)pmd09 VE are available, the 2022/23 interim season estimates were lower: 26% (among ≥ 18-year-olds) vs 76% (among ≥ 50-year-olds) in EN-EC and 28% vs 75% (among all ages) EU-PC [16,17]. The influenza vaccine component remained the same between these two seasons; however, circulating strains differed. While post-infection ferret antisera raised against the vaccine strain A/Victoria/2570/2019 had good recognition to circulating viruses, post-vaccination human sera showed lower reactivity [18]. The 2022/23 end-of-season overall results, as well as clade/genetic variant-specific results and birth cohort-specific VE, may help unravel the differences between these two seasons. Additionally, around 25% of all sequenced influenza A(H1N1)pdm09 viruses in DK-PC/DK-H and in EU-PC, and all 20 sequenced viruses in SC-H belonged to the A/Norway/25089/2022-like viruses.

The VE point estimates against influenza A(H3N2) among the three primary care and emergency care studies (DK-PC, EU-PC and EN-EC) over adult ages, at 36–42%, were slightly lower than the VE point estimates from Canada (58–59%) [19]. Among children, the primary care and emergency care study results presented here were higher at 62–70% compared with those in the Canadian study (47%). Authors in Canada noted a high proportion of T135K substitutions among those ≤ 25 years of age. Position 135 is a haemagglutinin (HA) glycosylation site associated with potential antigenic change [20]. Information on substitutions at this position is not available from all studies, but only two of the 444 sequenced EU-PC viruses and only one of 93 from the DK-H/PC studies, also in an individual aged < 25 years, harboured the T135K substitution. In EU-PC, 11% (51/444) of sequenced samples had a T135A substitution, which also involves the loss of the HA glycosylation site. All A(H3N2) viruses with available genetic information from the studies presented here belonged to the 3C.2a1b.2a.2 clade, but with varying genetic diversity within this clade.

In the DK-PC, DK-H, EN-EC and EU-PC studies, for which end-of-season 2021/22 influenza A(H3N2) VE estimates were available, the overall 2022/23 interim results against influenza A(H3N2) were higher for EN-EC and EU-PC studies (37% vs 28% and 44% vs 29%, for EN-EC and EU-PC, respectively, noting a difference in the reported age cohort for EN-EC) [16,17]. For DK-PC and DK-H, 2021/22 influenza A(H3N2) varied considerably by age group, particularly above and below 45 years of age, and cannot be directly compared with the interim 2022/23 A(H3N2) VE results in these studies [3]. However, VE was generally low (23% and 2% for DK-PC and DK-H, respectively), although sample size was also low. As the 2021/22 season was a long and late season in Europe, the 2021/22 A(H3N2) VE may have declined with time since vaccination, as reported by the EU-PC study, rendering the overall 2021/22 season A(H3N2) estimates not comparable to 2022/23 interim estimates. While reports suggest a good antigenic match between circulating and vaccine strains for A(H3N2) in 2022/23, there was considerable genetic diversity within the 3C.2a1b.2a.2 clade, and end-of-season and clade/genetic variant-specific results may help understand differences between sites.

Influenza B virus has had little circulation in Europe since the 2019/20 season. The observed VE against influenza B was high at ≥ 50%, with estimates among children at ≥ 87%. Influenza B VE is often high, as seen in the 2019/20 season in Canada, the United States, Denmark, Spain and in the EU-PC study [21-23]. All sequenced viruses belonged to the V1A.3a.2 clade and, as expected, no influenza B/Yamagata was detected among sequenced viruses. Recent B/Victoria viruses harbour substitutions at positions resulting in a phenotypic ‘reversion’ to viruses with similar antigenic properties to viruses circulating ≥ 50 years before [24]. Potential imprinting effects may explain differing VE by birth cohort and could be explored further if end-of-season sample size allows.

In general, across influenza (sub)types, particularly for influenza A(H3N2) and B, VE point estimates were high in children. While some confidence intervals overlapped between children’s and adults’ estimates, the point estimates were consistently higher among children across all influenza (sub)types in each study. LAIV is part of the routine childhood immunisation schedule in the UK and has been introduced in Ireland and Denmark in recent years. The use of LAIV could contribute to the age-specific differences in VE and these results indicate good performance of LAIV in this season. A further contribution to age-specific differences in VE is that routine childhood immunisation is targeted towards all children, including healthy children. In contrast, in young adults, immunisation is indicated mainly for those with underlying medical conditions, who may be at greater risk of influenza infection/hospitalisation.

The early start of the season in most European countries included in these six studies [5-7] resulted in higher incidence and greater precision for interim VE estimates than in other interim season estimates. However, due to the different circulation of influenza viruses across Europe, some studies had lower sample size for some subgroups in this interim analysis and results should be interpreted with caution. Each study used their own specific criteria to define whether a sample size was too small to attempt VE estimation. Sensitivity analyses were used to address potential small sample bias where appropriate. These studies are all observational in nature and residual confounding and bias may potentially be present.

### Conclusion

Vaccination remains a successful means of influenza prevention. Interim results from six European studies during the 2022/23 influenza season indicate a ≥ 27% and ≥ 50% reduction in disease occurrence among all-age influenza vaccine recipients for influenza A and B, respectively. Influenza VE point estimates were ≥ 50% against all influenza (sub)types in children, indicating a successful LAIV campaign. Influenza vaccination should continue to be promoted according to national guidelines in all European countries with ongoing influenza virus circulation.

Findings of the current study were presented as part of the Global Influenza Vaccine Effectiveness (GIVE) report to the WHO Vaccine Strain Selection Committee, held on 20–23 February 2023. In this meeting, the WHO recommendations for the 2023/24 Northern Hemisphere influenza vaccine viruses did not change for influenza B/Victoria, B/Yamagata or A(H3N2) [25]. For influenza A(H1N1)pdm09, the recommendation for the 2023–24 influenza vaccines changed to A/Victoria/4897/2022 (H1N1)pdm09-like virus for egg-based vaccines and A/Wisconsin/67/2022 (H1N1)pdm09-like viruses for cell-based vaccines. 

End-of-season influenza VE and genetic analyses may help understand observed differences in age as well as study-specific VE.
